# Genomic and phenotypic analysis of *Weissella cibaria* LB13201 and LB13206 isolated from Hanwoo (native Korean cattle) with antimicrobial and anti-inflammatory capability

**DOI:** 10.3389/fmicb.2026.1674601

**Published:** 2026-02-19

**Authors:** Min-Gyu Lee, Soo-Young Yum, Gyeong-Min Gim, Kyeong-Hyeon Eom, Do Young Jin, Jun-Seob Kim, Goo Jang

**Affiliations:** 1 LART Bio Inc., Gwangmyeong-si, Republic of Korea; 2 Department of Theriogenology and Biotechnology, College of Veterinary Medicine, Seoul National University, Seoul, Republic of Korea; 3 Department of Nano-Bioengineering, Incheon National University, Incheon, Republic of Korea; 4 Institute for New Drug Development, College of Life Science and Bioengineering, Incheon National University, Incheon, Republic of Korea

**Keywords:** anti-inflammatory activity, antimicrobial activity, genomic analysis, probiotic characteristics, safety assessment, *Weissella cibaria*

## Abstract

Probiotics are widely used in livestock to improve gut health, enhance productivity, and increase disease resistance. *Weissella* strains, particularly *W. cibaria,* have probiotic properties. However, research on the *Weissella* genus remains limited. This study investigated the genomic, functional, and probiotic characteristics of *W. cibaria* strains LB13201 and LB13206, which were isolated from the bovine vagina. Whole-genome sequencing revealed that the strains possess circular chromosomes with two (LB13206) or three (LB13201) plasmids, containing 2,253 and 2,311 coding sequences, respectively. Taxonomic classification confirmed its identity as *W. cibaria*. The analyzed strains exhibited tolerance to acidic environments and bile salts, along with auto- and co-aggregation capabilities, indicating potential for gut colonization and pathogen exclusion. Functional gene analysis identified carbohydrate-active enzymes, such as glycoside hydrolases and glycosyltransferases, essential for carbohydrate metabolism and food fermentation. Additionally, the presence of a Type III polyketide synthase gene and a terpene-precursor cluster suggests potential for bioactive metabolite production. Despite the absence of bacteriocin-encoding genes, *W. cibaria* LB13201 and LB13206 displayed antimicrobial activity against pathogens. Comprehensive safety assessments confirmed the absence of virulence factors, antibiotic resistance genes, hemolytic activity, or gelatinase production, supporting its safety profile. The strain also exhibited antioxidant and anti-inflammatory properties, suggesting additional health benefits. In conclusion, *W. cibaria* LB13201 and LB13206 are promising probiotic candidates for potential application in cattle feed additives.

## 1 Introduction

Probiotics are defined as “live microorganisms which, when administered in adequate amounts, confer a health benefit on the host” (FAO/WHO, 2002). The application of direct-fed microbials (DFM) in enhancing livestock health and performance has garnered significant interest within the industry. In the context of feedlot beef cattle, probiotics are primarily employed to promote overall health by preventing or alleviating ruminal acidosis, improving weight gain and feed conversion efficiency, and reducing the shedding of human pathogens (Uyeno et al., 2015; Retta, 2016). Probiotic strains have garnered considerable attention within both the food and pharmaceutical sectors due to their potential health benefits. Research is currently ongoing to explore these strains as therapeutic agents that may enhance growth performance, improve feed intake efficiency, optimize rumen fermentation, and bolster immune and antioxidant functions in cattle. Additionally, the adoption of probiotics in animal feed has seen a notable increase, resulting in enhancements in animal performance, digestion, overall productivity, and resilience against infectious diseases (Uyeno et al., 2015; Markowiak and Śliżewska, 2018).

Probiotics in cattle diets typically include various microbial strains, such as lactic acid bacteria (LAB), yeast, and spore-forming bacteria. These beneficial microbes enhance ruminal fermentation by stabilizing pH, reducing lactic acid accumulation, and balancing volatile fatty acids (Ogbuewu and Mbajiorgu, 2023). Probiotics are increasingly studied and used in the food industry to manage livestock intestinal infections and reduce antibiotic use in animal feed. This method helps to reduce the spread of antibiotic resistance and lowers the amount of antibiotics found in products from animals. Additionally, probiotics in cattle diets can enhance gut function, optimize feed efficiency, and improve overall health and productivity in ruminants (Patrone et al., 2021). Among LAB, the probiotic potential of *Weissella* species has only recently been studied in both humans and animals. *Weissella* strains, particularly *W. cibaria, W. confusa,* and *W. paramesenteroides*, have probiotic properties (Patrone et al., 2021). *Weissella* spp. are frequently detected in cow feces, vaginal tract, skin, and milk, suggesting they are well adapted to cattle (Patrone et al., 2021). *Weissella cibaria* is particularly notable owing to its antimicrobial, immune-enhancing, and antioxidant activities (Teixeira et al., 2021).

Before its use as a feed additive, its safety and functional properties must be assessed; probiotic safety evaluations include tests for antibiotic resistance, virulence genes, hemolytic activity, and toxic metabolite production, ensuring their suitability for animal consumption (Jang et al., 2021). Once deemed safe, *W. cibaria’s* functional characteristics should be assessed for livestock benefits. *W. cibaria* JW15 has shown antioxidant and anti-inflammatory effects *in vitro* (Yu et al., 2019a,b). *W. cibaria* CMU has demonstrated strong antimicrobial activity against oral pathogens (Yeu et al., 2021). However, research on bacteriocins produced by the *Weissella* genus remains limited.

This study analyzed the genome and phenotype of *W. cibaria* LB13201 and LB13206, isolated from the cattle vagina, to evaluate their probiotic potential and safety profile. Functional annotation has revealed the presence of carbohydrate metabolic enzymes, and comprehensive assessments have been carried out to evaluate gastrointestinal (GI) tolerance, auto-aggregation, co-aggregation, as well as antioxidant, anti-inflammatory, and antibacterial activities. Our findings contribute valuable insights for the selection of beneficial strains with specific genetic traits, with potential applications in animal nutrition.

## 2 Materials and methods

### 2.1 Isolation of LAB and bacterial growth culture

Hanwoo (native Korean cattle) was selected to isolate LAB. Before sampling, the external genitalia were disinfected, and vaginal samples were collected using sterile cotton swabs. Samples were immediately suspended in phosphate-buffered saline (PBS, pH 7.4) and stored at 4 °C until further processing. Serial dilutions were prepared using sterile 0.85% (w/v) NaCl, and aliquots from each dilution were plated on bromocresol purple (BCP)-de Man-Rogosa-Sharpe (MRS; BD Difco™, Franklin Lakes, NJ, United States) agar. The plates were incubated anaerobically at 37 °C for 20 h. Thereafter, yellow-colored colonies on BCP-MRS agar medium were selected from each sample and subcultured twice on fresh BCP-MRS agar medium. *W. cibaria* LB13201 and LB13206 were preserved in MRS broth containing 20% glycerol at −80 °C. For cultivation, strains were grown in MRS broth and incubated at 37 °C for 24 h.

The bacterial strains used in this study, *Lacticaseibacillus rhamnosus* ATCC 53103, *Bacillus subtilis* ATCC 6633, *Escherichia coli* ATCC 10536, *Staphylococcus aureus subsp. aureus* ATCC 6538, *Klebsiella oxytoca* ATCC 8724, *Salmonella enterica subsp. enterica* ATCC 14028, and *Listeria monocytogenes* ATCC 15313 were obtained from the Korean Collection for Type Cultures (Jeongeup, Korea). *Lacticaseibacillus rhamnosus* ATCC 53103 was cultured in MRS broth, while the other bacteria were cultured in Luria–Bertani broth (LB; Difco™, New Jersey, United States).

### 2.2 DNA extraction and whole-genome sequencing

Genomic DNA was isolated using the AccuPrep Genomic DNA Extraction kit (Bioneer, Daejeon, Korea). DNA concentration was quantified using the Qubit Flex Fluorometer (Thermo Fisher Scientific, MA, United States) with a Qubit dsDNA (double-stranded DNA) HS assay kit (Thermo Fisher Scientific).

A short-insert paired-end library (2 × 151 bp) for whole genome sequencing (Illumina iSeq 100 system) was prepared with 300 ng of genomic DNA using the Illumina DNA prep kit (Illumina, San Diego, United States). The sample was barcoded with indices from the Illumina DNA/RNA UD Indexes Set A (Illumina). The library was calculated with a Qubit flex Fluorometer using a Qubit dsDNA HS Assay Kit and sequenced using the iSeq 100 i1 Reagent (300-cycle) kit (Illumina).

A long-read library for whole genome sequencing (Oxford Nanopore MinION) was prepared as follows: 1 μg genomic DNA was repaired using the NEBNext FFPE DNA Repair Mix (New England Biolabs, MA, United States), followed by end-prep with the NEBNext Ultra II End Repair/dA-Tailing Module (New England Biolabs), and cleaned up using 1 × AMPure XP beads (Beckman Coulter, CA, United States). Barcode ligation was performed using NEB Blunt/TA Ligase Master Mix (New England Biolabs) and the Native Barcoding Kit 24 V14 (SQK-NBD114.24) (Oxford Nanopore Technologies, Oxford, UK), followed by purification with 0.4 × AMPure XP beads. Adapter ligation was performed using the Native Barcoding Kit 24 V14 ((SQK-NBD114.24), Oxford Nanopore Technologies) and NEBNext Quick Ligation Module (New England Biolabs). The library was purified with Long Fragment Buffer (Oxford Nanopore Technologies) and 0.4 × AMPure XP beads and subsequently quantified using a Qubit Flex Fluorometer with a Qubit dsDNA HS Assay Kit. Finally, long-read sequencing was performed using the MinION Flow Cell R9.4.1 (Oxford Nanopore Technologies).

### 2.3 Nucleotide sequencing of 16S rRNA gene

Identifying the bacterial species, PCR amplification of the 16S rRNA gene was conducted using a PCR premix (iNtRON Biotechnology, Seongnam, Korea) per the manufacturer’s instructions. The reaction mixture contained 20 ng of template DNA and the universal primers 27F (5′-AGA GTT TGA TCA TGG CTC AG-3′) and 1492R (5′-TAC GGY TAC CTT GTT ACG AC-3′). Thermal cycling involved an initial denaturation at 95 °C for 5 min, followed by 35 cycles of denaturation (94 °C, 30 s), annealing (57 °C, 30 s), and extension (72 °C, 1 min), concluding with a final elongation at 72 °C for 5 min. The resulting amplicons were purified using a QIAquick^®^ PCR Purification Kit (Qiagen, Hilden, Germany) and sequenced using a 3730XL DNA Analyzer (Thermo Fisher Scientific) at Macrogen (Seoul, Korea). Sequence similarity searches were performed against the NCBI database using the Basic Local Alignment Search Tool (BLAST). For phylogenetic analysis, multiple sequence alignments were generated using ClustalX v2.1 (Larkin et al., 2007), and evolutionary trees were constructed using MEGA v11 (Saitou and Nei, 1987).

### 2.4 Genome assembly and functional annotation

Genome assembly was performed using Unicycler, a hybrid assembler that integrates short-read Illumina data with long-read sequencing ONT nanopore data. The genome annotation was performed using Rapid Annotations with Subsystems Technology (RAST) (Aziz et al., 2008). Additionally, the genome was analyzed for carbohydrate-active enzymes (CAZymes) using the dbCAN3 tool (Zheng et al., 2023), an automated platform for annotating CAZyme families based on the Carbohydrate-Active EnZymes database (Cantarel et al., 2009). To ensure high confidence, the annotation integrated results from three tools (HMMER, DIAMOND and dbCAN-sub). Only hits identified by all three tools were considered valid CAZymes. Chromosome and plasmid visualizations were generated using the Proksee server (Grant et al., 2023).

### 2.5 Species confirmation with average nucleotide identity analysis

Species identification was performed using the Bactopia v3.1.0 pipeline, incorporating the Genome Taxonomy Database (GTDB) workflow (Chaumeil et al., 2020). This method compares the genomic sequences of *W. cibaria* LB13201 and LB13206 against a comprehensive database of microbial genomes to determine taxonomic classification. Key genomic characteristics, including sequence similarity, gene composition, and phylogenetic relationships, were analyzed to identify the most closely related species within the GTDB. Additionally, the Average Nucleotide Identity (ANI) was evaluated across *W. cibaria* genomes retrieved from the NCBI RefSeq database. To evaluate species-level identity, ANI analysis was performed against *W. cibaria* genomes retrieved from the NCBI RefSeq database. A total of 50 reference genomes were selected to encompass the genomic diversity of the species, representing various ecological niches. Pairwise ANI values were calculated using FastANI v1.33 (Jain et al., 2018).

### 2.6 Exploration of genes encoding bacteriocins and secondary metabolite prediction

Screening for bacteriocin-encoding genes was performed using Bagel4 v.1.2 (van Heel et al., 2018), which detects and classifies bacteriocin gene clusters based on homology searches against curated databases and motif analyses. Additionally, the annotation and analysis of secondary metabolite biosynthesis gene clusters were performed using antiSMASH v8.0.1 (Blin et al., 2023).

### 2.7 *In silico* safety assessment

The whole genome sequence of *W. cibaria* LB13201 and LB13206 was assessed as previously described by Lee et al. (2024). Pathogenic potential was assessed using PathogenFinder v1.1 (Cosentino et al., 2013) to predict its ability to infect human hosts. Res-Finder v.4.6.0,^1^ Comprehensive Antibiotic Resistance Database (CARD),^2^ RGI v.6.0.3, and Virulence-Finder 2.0^3^ were used to identify antibiotic resistance genes (ARG) and virulence genes. Homologous sequences to *E. coli*, *Listeria*, *S. aureus*, and *Enterococcus* virulence genes were screened with a minimum threshold of 90% for percent identity (%ID) and 60% for minimum length.

### 2.8 Polymerase chain reaction detection of antimicrobial resistance genes and potential virulence factors

PCR was performed using forward and reverse primer pairs, alongside standard PCR reagents in a 10 μL reaction mixture. Each reaction contained 10 ng genomic DNA, 0.5 pM of each primer, 1 μL 10 × PCR buffer, 250 μM dNTPs (2.5 mM each), and 0.25 units of i-StarTaq DNA Polymerase (5 units/μL) (iNtRON Biotechnology, Seongnam, Korea). For LB13201 and LB13206, target genes were identified through PCR using gene-specific primers, which were designed based on sequences previously studied, and their specificity was confirmed using antibiotic-resistant reference strains (Supplementary Table S1). PCR of antibiotic resistance and virulence factor genes in *W. cibaria* LB13201 and LB13206 was performed using the following cycling conditions: Amplification included an initial denaturation (95 °C, 5 min), 35 cycles of denaturation (94 °C, 30 s), annealing (detailed in Supplementary Table S1), elongation (72 °C, 1.5 min), and a final extension (72 °C, 5 min). PCR products were examined via electrophoresis on 1.2% (w/v) agarose gels prepared using Tris-acetate EDTA buffer (40 mM Tris-acetate, 1 mM EDTA, pH 8.0).

### 2.9 Antibiotic susceptibility test using E-test

The antibiotic susceptibility of *W. cibaria* LB13201 and LB13206 was evaluated using E-test strips (ETEST®, bioMérieux, Marcy-l’Étoile, France) as described previously by Lee et al. (2024). The bacterial inoculum was prepared by adjusting to 0.5 McFarland. The adjusted culture was streaked across the Mueller Hinton Agar (Thermo Fisher Scientific) using a sterile cotton swab. Following inoculation, the E-test strip was placed onto the agar surface, and the plate was subsequently incubated at 37 °C for 48 h. Minimum inhibitory concentration (MIC) was determined based on cut-off values recommended by the European Food Safety Authority (EFSA et al., 2018).

### 2.10 Hemolysin and gelatinase activity

Hemolysin and gelatinase activities were assessed using established methods. Hemolysin activity was tested on blood agar plates (Biozoa Biological Supply Company, Seoul, Korea) supplemented with 5% defibrinated sheep blood. The plates were incubated at 37 °C for 24 h. The presence of clear zones around colonies indicated β-hemolysin production. *Staphylococcus aureus* ATCC 6538 was used as a positive control. Gelatinase activity was evaluated by inoculating LAB onto freshly prepared MRS with gelatin from bovine skin (Sigma-Aldrich, St. Louis, MO, United States). After overnight incubation at 37 °C, the plates were cooled in an ice bath for 30 min. Gelatin liquefaction indicated a positive result. *Lacticaseibacillus monocytogenes* ATCC 15313 was used as a positive control.

### 2.11 Cell cytotoxicity assay

The cytotoxicity of *W. cibaria* LB13201 and LB13206 was evaluated using RAW 264.7 cells, following the method described previously (Lee et al., 2024). RAW 264.7 cells were seeded in a 96-well plate at a density of 5 × 10^4^ cells/well and incubated at 37 °C with 5% CO_2_ for 20 h. Probiotic samples at multiplicity of infection (MOI) 50, 100, and 200 were added, while the negative control group received no treatment (MOI 0). Cells were further incubated for 24 h, and cytotoxicity was assessed using the CELLOMAX™ Viability Assay kit (Precaregene, Gyeonggi-do, Korea). After replacing the medium, the plate was incubated at 37 °C with 5% CO_2_ for 1 h. Cell viability was determined by measuring absorbance at 450 nm using an Epoch 2 microplate spectrophotometer. Blank wells without cells served as controls.

### 2.12 Enzyme production

Enzyme production was analyzed using the API ZYM kit (BioMérieux). The *W. cibaria* LB13201 and LB13206 bacterial culture was centrifuged at 14,240 × *g* for 5 min at 4 °C, and the cell pellet was resuspended in PBS. In total, 65 μL of the cell suspension (10^6^ CFU/mL) was inoculated into each cupule and incubated at 37 °C for 4 h. Following incubation, ZYM A and ZYM B reagents were sequentially added to each cupule. Enzyme production was determined by measuring substrate hydrolysis, indicated by a color change.

### 2.13 Determination of acid and bile tolerance

The acid and bile salt tolerance assays were performed according to previously described methods, with minor modifications (Jeong et al., 2023). For acid tolerance, the bacterial culture, grown overnight in MRS broth, was adjusted to pH 2.5, 3.0, and 4.0 using HCl in a 100 mM glycine-HCl buffer. The suspension was incubated at 37 °C for 2 h. After incubation, strains were plated on an MRS agar plate. For bile tolerance, the bacterial culture was supplemented with or without 0.3% (w/v) bovine and ovine bile (Sigma), adjusted to a final concentration of 0.5% (v/v). The mixture was subsequently incubated at 37 °C for 16 h. Survival rate was calculated using Equation (1):


(1) $$\text{Survival rate}=\frac{\text{Final logCFU}\;{\mathrm{ml}}^{-1}}{\text{Initial logCFU}\;{\mathrm{ml}}^{-1}}\times 100$$

### 2.14 Auto and co-aggregation assay

The auto-aggregation assay was performed as previously described (Kos et al., 2003). Overnight cultures of *W. cibaria* LB13201 and LB13206 grown in MRS broth were harvested via centrifugation at 4,000 rpm for 15 min, washed twice with PBS, and resuspended in PBS to achieve an OD_600_ of 1. The cell suspension was vortexed for 10 s and aliquoted into 4 mL samples. Absorbance at 600 nm was measured immediately (*t* = 0) and after 1, 2, 3, and 4 h of incubation at 37 °C. At each time point, 100 μL of the upper suspension was transferred into a new tube containing 3.9 mL of PBS, and the absorbance was recorded. The percentage of auto-aggregation was calculated using Equation (2):


(2) $$\text{Auto}-\text{aggregation}\;(\%)=(1-\frac{A_{t}}{A_{0}})\times 100$$

where *A_t_* represents the absorbance measured at a specific time (1, 2, 3, or 4 h), and *A*_0_ represents the absorbance at time zero (*t* = 0).

*E. coli* ATCC 10536, *S. aureus* ATCC 6538, *K. oxytoca* ATCC 8724, *S. enterica* ATCC 14028, and *L. monocytogenes* ATCC 15313 were used as test pathogens for the co-aggregation assay. Bacterial suspensions were adjusted to an OD_600_ of 0.5, and 2 mL of *W. cibaria* LB13201 and LB13206 was mixed with an equal volume of the pathogen. The mixtures were vortexed and incubated at 37 °C. Control tubes contained 4.0 mL of individual bacterial suspensions. The OD_600_ of each suspension was measured at t = 0 and after 1, 2, 3, and 4 h by transferring 100 μL from the upper layer into a new tube containing 3.9 mL PBS. Co-aggregation percentages were calculated according to the method described previously by Handley et al. (1987).

### 2.15 Antioxidant activity

Antioxidant activity was assessed for *W. cibaria* LB13201 and LB13206 using the OxiTec™ DPPH Antioxidant Assay Kit (BIOMAX, Seoul, Korea). To prepare the bacterial culture, a seed culture was established by growing the strains in MRS broth at 37 °C for 20 h. This pre-culture was then subcultured into fresh MRS medium at a 1% (v/v) inoculation and incubated at 37 °C for 16 h. Following growth, bacterial cells were removed by centrifugation at 21,000 × g for 5 min. The supernatant of LB13201 and LB13206 was mixed with DPPH solution and assay buffer in a 96-well plate following the manufacturer’s protocol. After a 30-min incubation in the dark at room temperature, absorbance was measured at 517 nm. The radical scavenging activity of LAB was compared to that of Trolox (100 μg/mL).

### 2.16 Anti-inflammatory activity assay

RAW 264.7 cells were seeded in 6-well plates at a density of 5 × 10^5^ cells per well and incubated at 37 °C with 5% CO₂ for 20 h. The cells were subsequently treated with *W. cibaria* LB13201 and LB13206 pellets, as detailed in Section 2.5.4. For the positive control, RAW 264.7 cells were treated with 1 μg/mL lipopolysaccharide (LPS). Nitric oxide (NO) secretion was quantified using the NO Plus Detection kit (iNtRON Biotechnology). Additionally, 1 μM dexamethasone was used as an additional positive control. Following treatment, cells were incubated for 2 h, followed by the addition of 1 μg/mL LPS and further incubation at 37 °C for 24 h.

### 2.17 Growth inhibition of pathogen bacteria

*W. cibaria* LB13201 and LB13206 were inoculated into MRS broth at a final concentration of 1% (v/v) and incubated at 37 °C for 24 h. The cell-free supernatant (CFS) was obtained via centrifugation, followed by filtration via a sterile 0.2-μM filter (Sartorius, Goettingen, Germany). Subsequently, 40 μL CFS and 160 μL LB medium were added to each well, and bacterial cultures (*E. coli* ATCC 10536, *S. aureus* ATCC 6538, *K. oxytoca* ATCC 8724, *S. enterica* ATCC 14028, and *L. monocytogenes* ATCC 15313) were inoculated at a final 1% (v/v) concentration. In the control group, 40 μL MRS and 160 μL LB medium were added to each well, followed by bacterial culture inoculation at a final concentration of 1% (v/v) and incubated at 37 °C for 24 h. The OD_600_ was measured every 4 h using an Epoch 2 microplate spectrophotometer (Agilent Technologies, Inc., California, United States).

### 2.18 Minimum inhibition concentration assay

The MIC of the CFS was determined following the Clinical and Laboratory Standards Institute guidelines (CLSI, 2018), with minor modifications. Before the assay, the CFS was prepared in the same way as that described in section 2.16. For the initial concentration, 100 μL of undiluted CFS was mixed with 100 μL of sterile Mueller–Hinton broth (MHB, Thermo Fisher Scientific), and subsequent two-fold serial dilutions were prepared in MHB. The assay was conducted in 96-well microtiter plates by combining 100 μL of each CFS dilution with 10 μL of bacterial inoculum to achieve a final of 1 × 10^4^ CFU/mL. Control included a growth control (inoculum in MHB) and a negative sterility control (MHB only). Following incubation at 37 °C for 24 h, the MIC was defined as the lowest CFS concentration that completely inhibited visible bacterial growth.

### 2.19 Statistical analyses

All results were presented as the mean ± standard deviation from three independent experiments. Statistical analysis was performed using one-way analysis of variance followed by Dunnett’s multiple comparisons test. All analyses were performed using GraphPad Prism version 9.0.0 for Windows (GraphPad Software, Boston, Massachusetts, United States^4^).

## 3 Results

### 3.1 Identification and genomic characteristics of LB13021 and LB13206

**Figure 1:**
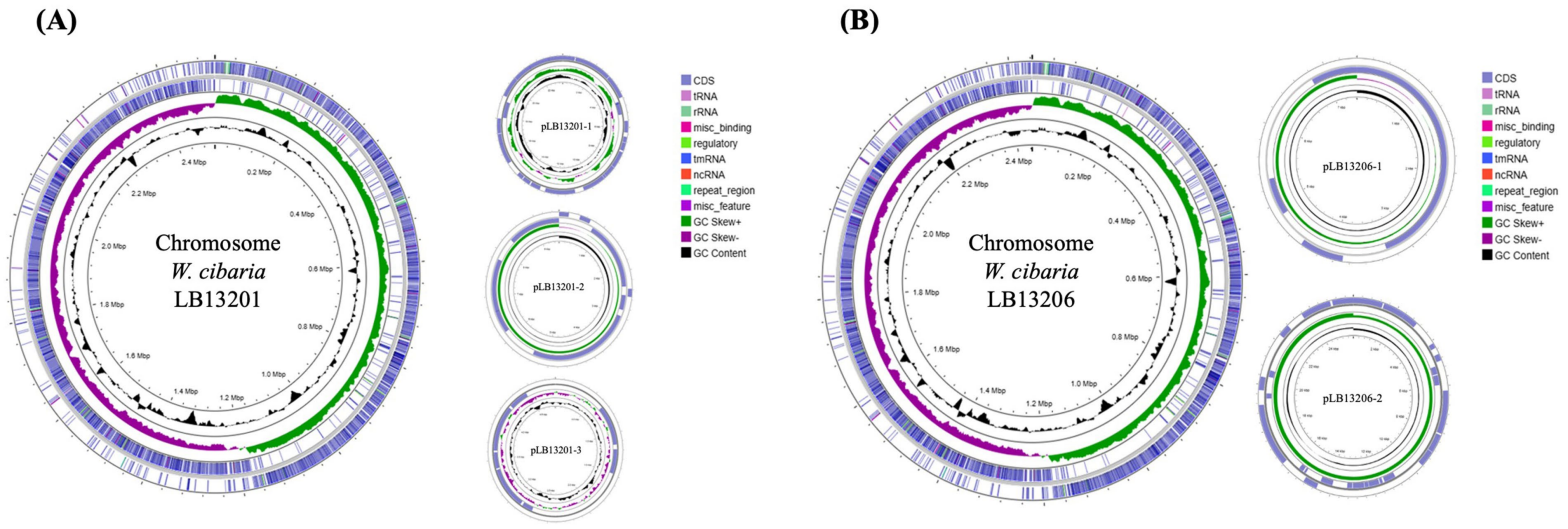
Circular genome map and annotation of *W. cibaria* LB13201 and LB13206. The genome atlas includes gene annotation features and GC skew analysis, generated using CGView 1.0. **(A)** *W. cibaria* LB13201; **(B)** *W. cibaria* LB13206.

Phylogenetic tree analysis indicated that the selected isolate belongs to *W. cibaria* (Supplementary Figure S1). The *W. cibaria* LB13201 comprises a circular chromosome and three plasmids, whereas *W. cibaria* LB13206 contains a circular chromosome and two plasmids, as illustrated in Figure 1. Both genomes span 2.44 Mb and contain 28 rRNA genes and 88 tRNA genes. However, *W. cibaria* LB13201 contains 1 tmRNA gene, whereas LB13206 contains 3. Regarding protein-coding sequences, LB13201 contains 2,311, while LB13206 contains 2,253 (Table 1). RAST annotation assigned functions to 752 coding sequences in LB13201 and 467 in LB13206, providing insights into the biological functions and metabolic pathways of these strains. Additionally, ANI analysis confirmed species-level identity. Taxonomic classification using the GTDB placed *W. cibaria* LB13201 and LB13206 in the genus *Weissella*, order *Lactobacillales*, and family *Lactobacillaceae*. To further assess species-level identity, FastANI analysis was performed against publicly available RefSeq genomes. LB13201 exhibited the highest ANI of 99.63% with *W. cibaria* AM113-89, while the lowest was 96.41% with *W. cibaria* FBL5. Similarly, LB13206 showed the highest ANI of 99.47% with *W. cibaria* CBA3636, and the lowest ANI was 96.33% with *W. cibaria* 7.8.34. All ANI values for both strains were above the 95% species-level threshold, supporting their classification within the *W. cibaria* species and indicating a consistent level of genomic similarity among strains. Since all ANI values exceeded the 95% species demarcation threshold, these findings confirm that both isolates belong to the *Weissella cibaria* species.

**Table 1:** Genomic characteristics of *W. cibaria* LB13201 and LB13206.

Feature	Chromosome		Plasmid				
	LB13201	LB13206	pLB13201-1	pLB13201-2	pLB13201-3	pLB13206-1	pLB13206-2
Size (bp)	2,443,905	2,443,905	23,510	9,542	4,818	25,947	7,412
GC content (%)	45.06	45.02	39.11	37.63	36.76	38.69	38.17
Protein-coding genes (CDS)	2,311	2,253	29	9	7	30	4
rRNA	28	28	0	0	0	0	0
tRNA	88	88	0	0	0	0	0
ncRNA	3	2	0	0	0	0	0
tmRNA	1	3	0	0	0	0	0

### 3.2 *In silico* safety evaluation

The analysis of ARG using the RGI program identified two strict hits associated with vancomycin resistance (vanT and vanY) in both *W. cibaria* strains (Table 2). However, the percent identity of these genes to the CARD reference sequences was low, ranging from 33.08 to 34.95%. Since these values are well below the generally accepted threshold of 70–80% for functional prediction, they were not considered to be functional resistance genes. Consistently, no significant ARG was detected when filtering for hits with >70% identity. Furthermore, comparative analysis with four pathogenic genomes revealed no virulence genes in *W. cibaria* LB13201 and LB13206, suggesting that these genes do not pose a safety concern. PathogenFinder further assessed the potential pathogenicity of *W. cibaria* LB13201 and LB13206 and detected no genes associated with pathogenic families. Most results aligned with non-pathogenic species, including *Lactobacillus casei*, *Streptococcus thermophilus*, *Lactobacillus kimchii*, *Leuconostoc mesenteroides*, and *Leuconostoc citreum*. The analysis yielded a pathogenicity score of 0.15 and 0.16, respectively, supporting the classification of *W. cibaria* LB13201 and LB13206 as a safe strain.

**Table 2:** Antibiotic resistance gene analysis of *W. cibaria* LB13201 and LB13206.

Strains	RGI criteria	AMR gene family	Drug class	% identity matching region
LB13201	Strict	vanT gene in the vanG cluster	Glycopeptide antibiotic	33.33
	Strict	vanY gene in the vanB cluster	Glycopeptide antibiotic	34.95
LB13206	Strict	vanT gene in the vanG cluster	Glycopeptide antibiotic	33.08
	Strict	vanY gene in the vanB cluster	Glycopeptide antibiotic	34.95

### 3.3 Identification of genes encoding bacteriocins and secondary metabolites

**Figure 2:**
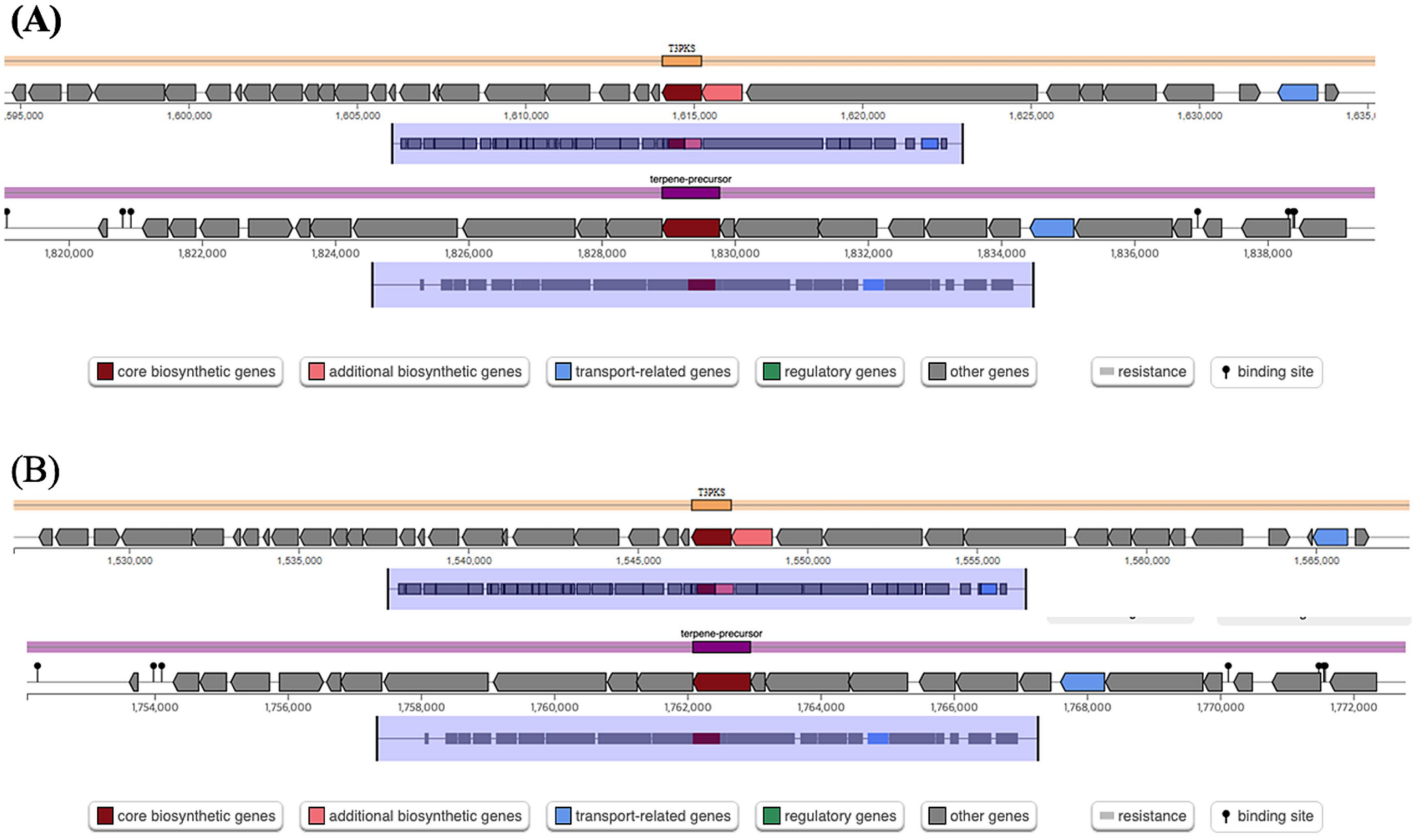
Secondary metabolite biosynthesis gene clusters identified in *W. cibaria* strains. **(A)** T3PKS and terpene-precursor biosynthetic gene clusters located at nucleotide positions 1,594,064–1,635,230 and 1,818,911–1,839,774, respectively, in *W. cibaria* LB13201; **(B)** T3PKS and terpene-precursor biosynthetic gene clusters located at positions 1,526,593–1,567,759 and 1,752,085–1,772,948, respectively, in *W. cibaria* LB13206.

No bacteriocin-encoding genes were identified in *W. cibaria* LB13201 and LB13206. However, secondary metabolite biosynthesis gene clusters, particularly the Type III polyketide synthase (T3PKS) and terpene-precursor cluster, were detected on its chromosome (Figure 2). In *W. cibaria* LB13201, the T3PKS biosynthetic gene cluster was located between nucleotide positions 1,594,064 and 1,635,230, encompassing 41,166 bp. In *W. cibaria* LB13206, the corresponding T3PKS cluster was located between positions 1,526,593 and 1,567,759, spanning 41,167 bp. The terpene-precursor biosynthetic gene cluster in *W. cibaria* LB13201 was located between positions 1,818,911 and 1,839,774, with a total length of 20,864 bp. Similarly, in *W. cibaria* LB13206, the cluster was found between positions 1,752,085 and 1,772,948, also spanning 20,864 bp.

### 3.4 Annotation of carbohydrate-active enzymes and subsystems

**Figure 3:**
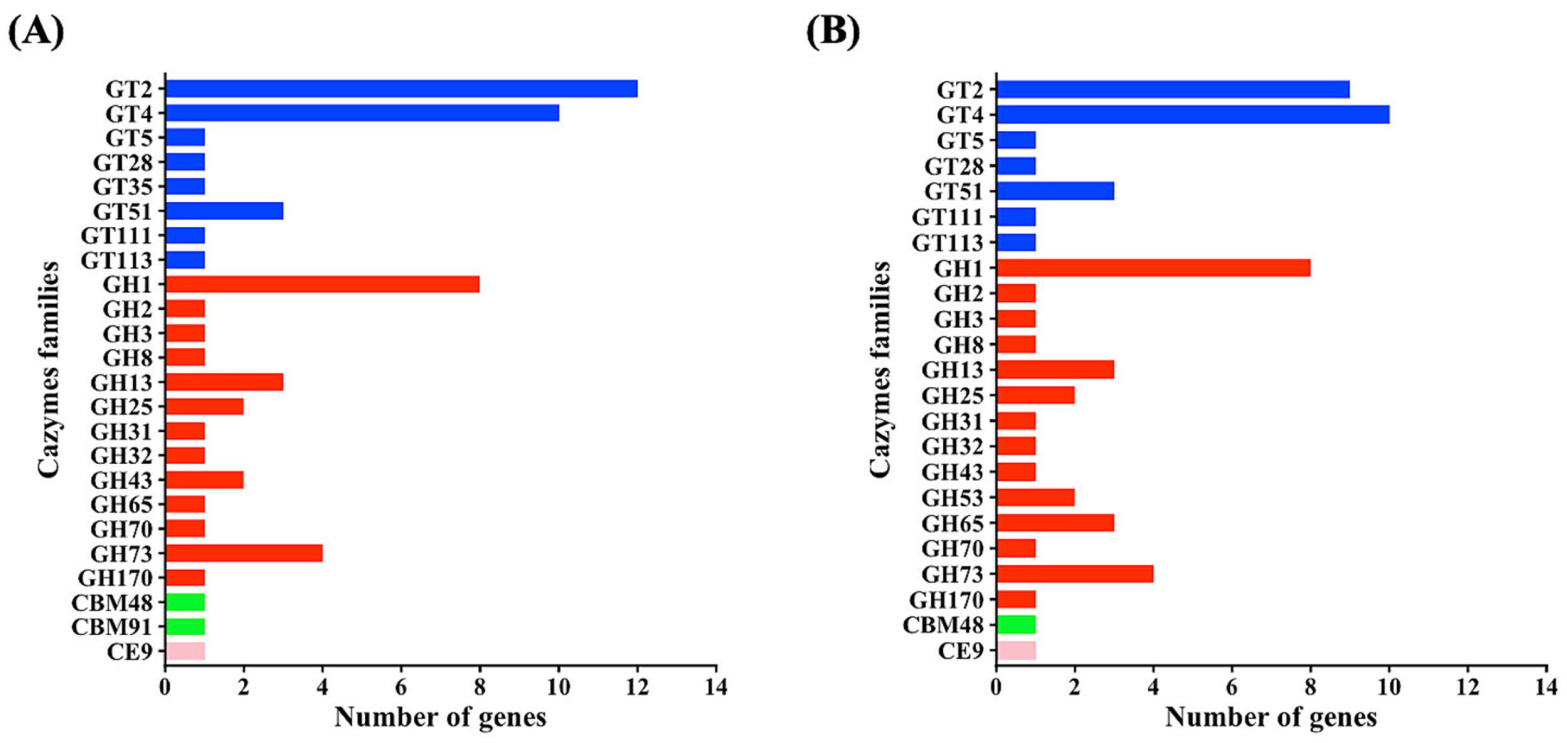
Distribution of CAZyme families and associated gene counts: red, GH; blue, GT; green, CBM; pink, CE. **(A)** Identified in the *W. cibaria* LB13201 genome and **(B)** identified in the *W. cibaria* LB13206 genome.

The *W. cibaria* LB13201 and LB13206 gene analysis revealed that most genes associated with its metabolic activity are involved in carbohydrate metabolism and transport. To confirm their functional roles, these genes were annotated using the dbCAN3 identified with all three tools (HMMER, DIAMOND, and dbCAN-sub). The analysis identified CAZyme domain sequences across five major families: glycosyltransferases (GT), glycoside hydrolases (GH), carbohydrate-binding modules (CBM), and carbohydrate esterases (CE) (Figure 3). In *W. cibaria* LB13201, GTs were the most abundant CAZyme family, represented by 30 genes distributed across 8 subfamilies, followed by GHs with 27 genes across 13 subfamilies; additionally, two CBMs and one CE were identified. Conversely, in *W. cibaria* LB13206, GHs were the predominant family, comprising 29 genes distributed across 14 subfamilies, followed by GTs with 26 genes across 7 subfamilies, along with one CBM and one CE. A comparative analysis of the subsystem annotation is presented in Supplementary Figure S2. The highest number of features was observed in the category protein metabolism, followed by amino acids, carbohydrates, nucleosides and nucleotides, and cofactors/vitamins. Both *W. cibaria* LB13201 and LB13206 exhibited similar profiles, with additional contributions from fatty acid and lipid metabolism, respiration, stress response, and defense mechanisms.

### 3.5 Acid and bile tolerance

The tolerance of probiotic strains to the harsh GI tract conditions is crucial for their probiotic effectiveness (Lee et al., 2012). In this study, Table 3 presents the tolerance of *W. cibaria* LB13201, *W. cibaria* LB13206, and *L. rhamnosus* ATCC 53103 to gastric and intestinal conditions. The survival rate of *W. cibaria* LB13201 at pH 2.5, 3.0, and 4.5 was 5.0, 55.0, and 89.4%, respectively, whereas *W. cibaria* LB13201 was 33.4, 57.0, and 87.2%, respectively. In bile salt conditions, the survival rates were 71.7% for LB13201 and 81.9% for LB13206. Compared to *L. rhamnosus* ATCC 53103, *W. cibaria* LB13201 and LB13206 showed limited resistance to low pH, maintaining over 80% growth only at pH 3.

**Table 3:** Gastrointestinal tolerance characteristics of *W. cibaria* LB13201 and LB13206.

Strain	pH 2.5 (%)	pH 3.0 (%)	pH 4.0 (%)	0.3% bile salt (%)
*W. cibaria* LB13201	5.0 ± 1.3	55.0 ± 0.7	89.4 ± 0.4	71.7 ± 0.6
*W. cibaria* LB13206	33.4 ± 0.4	57.0 ± 0.7	87.2 ± 0.4	81.9 ± 0.6
*L. rhamnosus* ATCC 53103	54.8 ± 5.0	99.8 ± 0.1	99.8 ± 0.1	91.6 ± 0.3

### 3.6 Auto- and co-aggregation activity

**Figure 4:**
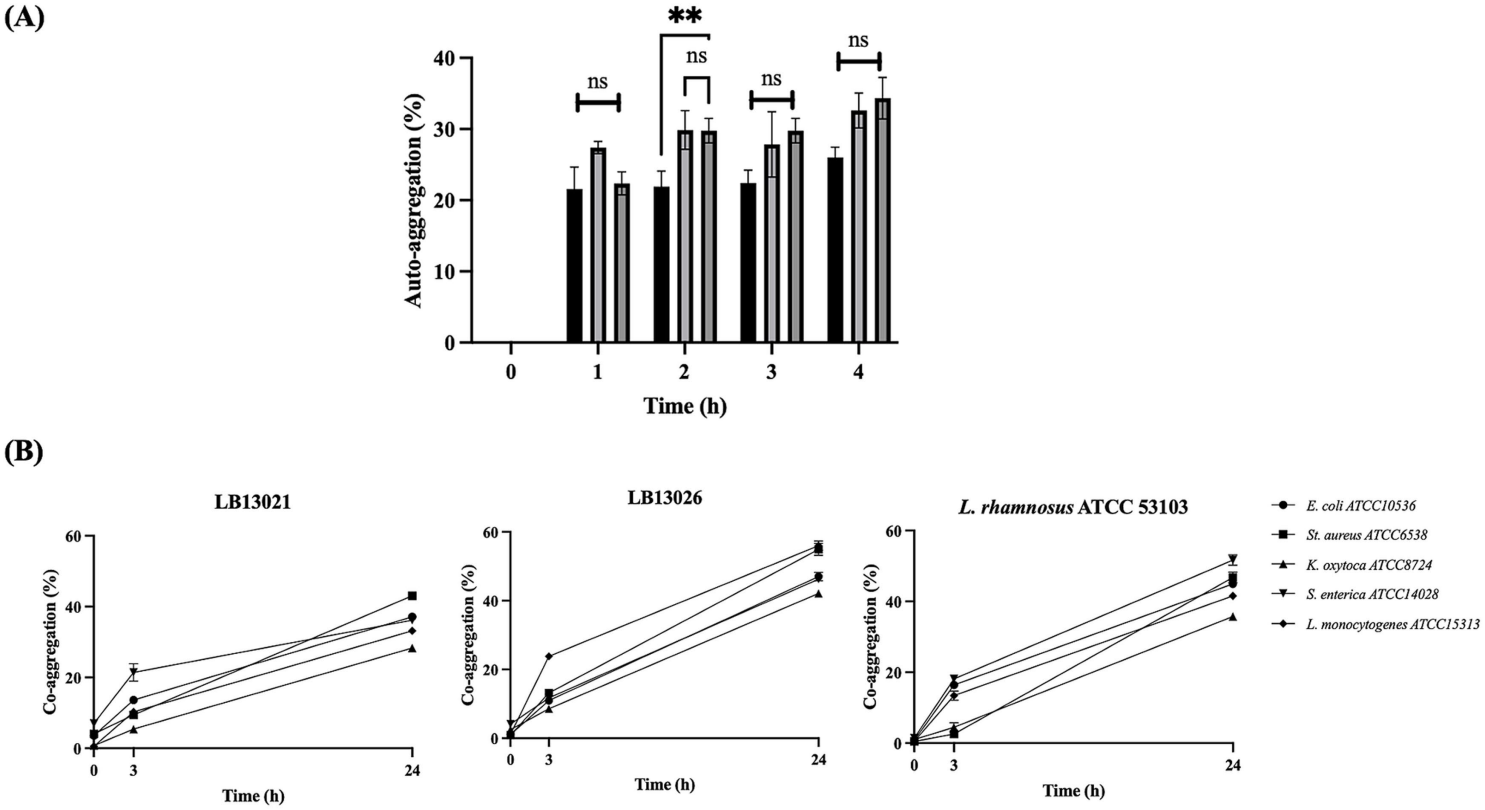
Auto-aggregation and co-aggregation abilities. **(A)** Auto-aggregation; **(B)** Co-aggregation of *W. cibaria* LB13201, LB13206, and *L. rhamnosus* ATCC 53103 with selected pathogens. Asterisks show significant differences among groups at 0.01 (*n* ≥ 3) via one-way ANOVA and Dunnett’s test. **p < 0.01 (*n* ≥ 3) via one-way ANOVA and Dunnett’s test.

The auto-aggregation of *W. cibaria* LB13201 exhibited a lower level of auto-aggregation compared to *L. rhamnosus* ATCC 53103, whereas *W. cibaria* LB13206 was similar to that of *L. rhamnosus* ATCC 53103 (Figure 4A). As shown in Figure 4B, when compared with *L. rhamnosus* ATCC 53103, which showed a co-aggregation percentage of 45.0% with *E. coli* ATCC 10536, *W. cibaria* LB13201 displayed a slightly lower value of 37.1%, whereas LB13206 reached a higher level of 47.0%. Against *K. oxytoca* ATCC 8724, LB13201 exhibited 28.3% co-aggregation, which was lower than the 35.8% observed for *L. rhamnosus* ATCC 53103, while LB13206 showed a higher interaction at 42.1%. For *S. aureus* ATCC 6538, LB13201 demonstrated 43.0% co-aggregation, close to the 46.8% of *L. rhamnosus* ATCC 53103, whereas LB13206 showed a markedly higher value of 54.9%. In *S. enterica* ATCC 14028, LB13201 showed 36.2%, which was lower than the 51.7% of *L. rhamnosus* ATCC 53103, while LB13206 exhibited 46.2%, remaining comparable to the reference strain. With *L. monocytogenes* ATCC 15313, LB13201 reached 33.2%, which was lower than the 41.6% of *L. rhamnosus* ATCC 53103, whereas LB13206 displayed the highest value at 56.1%. *W. cibaria* LB13201 exhibited a co-aggregation ability comparable to *L. rhamnosus* ATCC 53103 only against *S. aureus* ATCC 6538. In contrast, LB13206 demonstrated a co-aggregation level similar to *L. rhamnosus* ATCC 53103 with *S. enterica* ATCC 14028, whereas with the other pathogens, it showed higher co-aggregation.

### 3.7 Antioxidant activity

**Figure 5:**
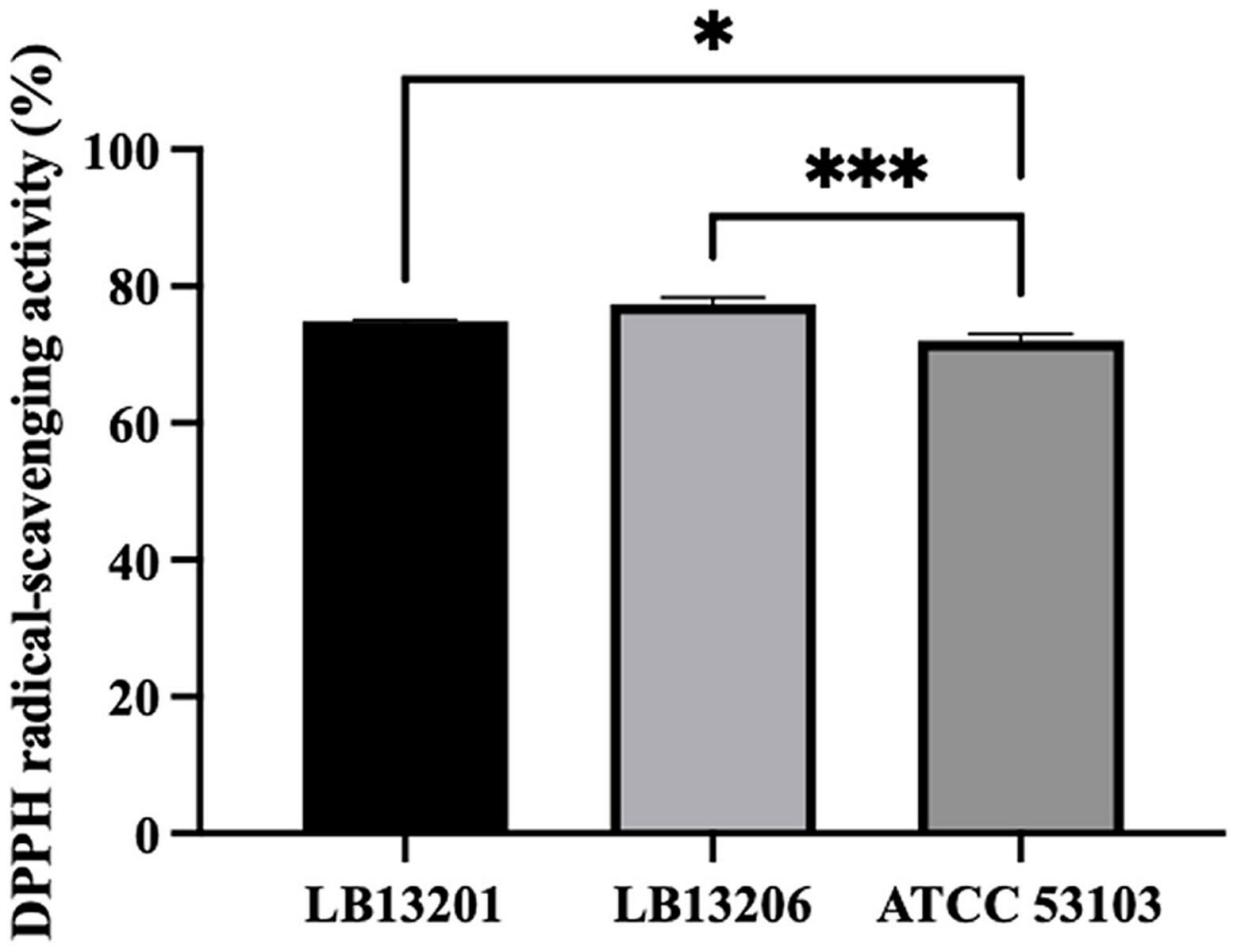
DPPH radical scavenging activity of *W. cibaria* strains. Black bar, *W. cibaria* LB13201; gray bar, *W. cibaria* LB13206; dark gray bar, *L. rhamnosus* ATCC 53103. Asterisks show significant differences among groups at 0.001 (*n* ≥ 3) via one-way ANOVA and Dunnett’s test. ***p < 0.001 (*n* ≥ 3) via one-way ANOVA and Dunnett’s test.

The DPPH radical scavenging activity of the tested strain was assessed and compared to the antioxidant activity of *W. cibaria* LB13201, *W. cibaria* LB13206, and *L. rhamnosus* ATCC 53103, demonstrating antioxidant activities of 74.9, 77.3, and 72.1%, respectively, highlighting the high DPPH radical scavenging potential of LB13201 and LB13206 (Figure 5).

### 3.8 Cell cytotoxicity and anti-inflammatory activity

**Figure 6:**
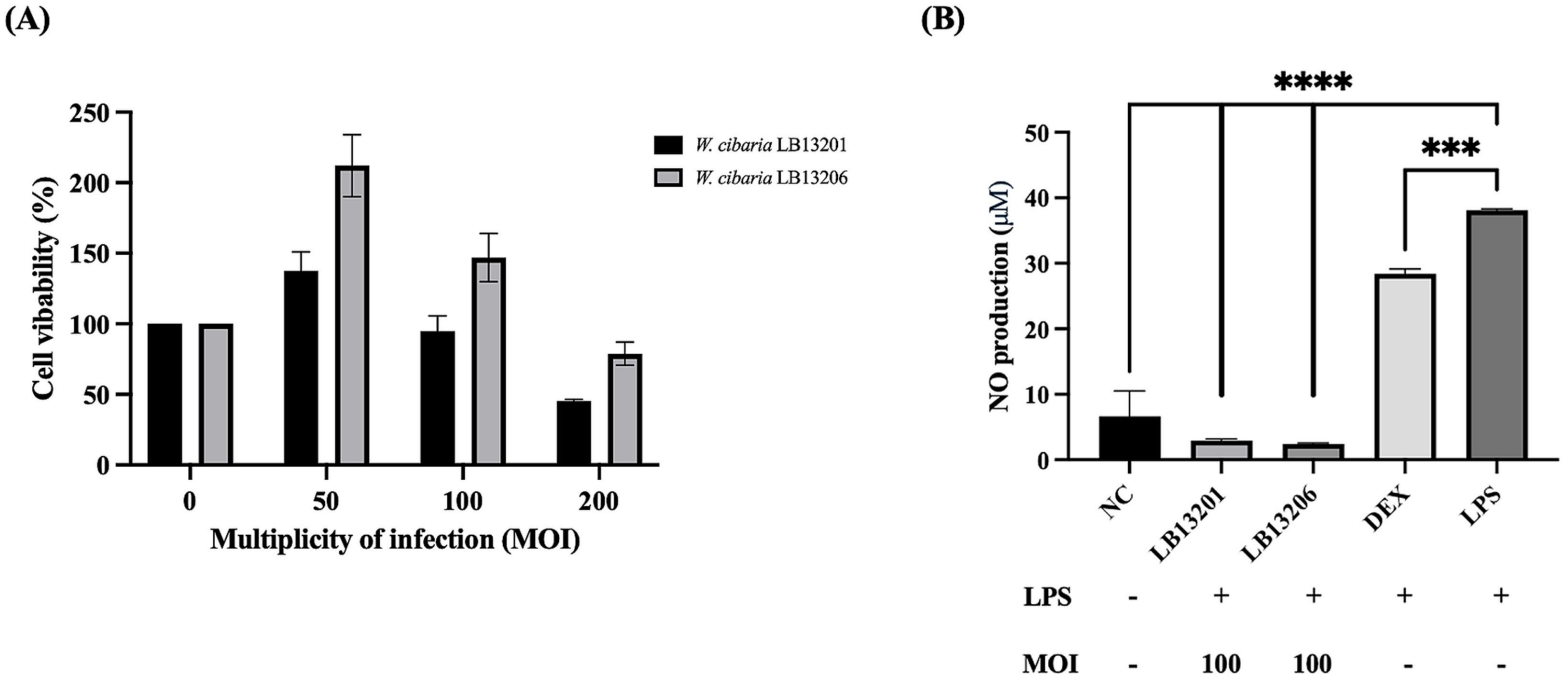
Cytotoxicity and nitric oxide (NO) production in RAW 264.7 macrophages treated with *W. cibaria* strains at a multiplicity of infection (MOI) of 100. **(A)** Cytotoxicity *of W. cibaria* LB13201 and LB13206 toward RAW 264.7 cells. Different letters show significant differences among groups at *p* < 0.05 using one-way ANOVA and Dunnett’s test; **(B)** NO production from RAW 264.7 cells treated with *W. cibaria* LB13201 and LB13206. Different letters among groups represent significant differences at *p* < 0.05. Asterisks show significant differences among groups at 0.001 (*n* ≥ 3) via one-way ANOVA and Dunnett’s test. ****p < 0.0001 (*n* ≥ 3) via one-way ANOVA and Dunnett’s test.

*W. cibaria* LB13201 and LB13206 showed no cytotoxicity in all treated groups, when examined in RAW 264.7 cells (Figure 6A). The anti-inflammatory activity of *W. cibaria* LB13201 and LB13206 on RAW 264.7 macrophage was examined. When 1 μg/mL LPS was added, 38.2 μM NO was produced. When 1 μM dexamethasone, a representative chemical that relieves inflammation, was added to RAW 264.7 cells, NO production significantly decreased to 29.1 μM. Similarly, treatment with *W. cibaria* LB13201 and LB13206 reduced NO levels by 2.7 μM and 2.3 μM, respectively (Figure 6B), indicating that *W. cibaria* LB13201 and LB13206 have considerable anti-inflammatory activity.

### 3.9 Growth inhibition activity and minimum inhibition concentration of CFS

**Figure 7:**
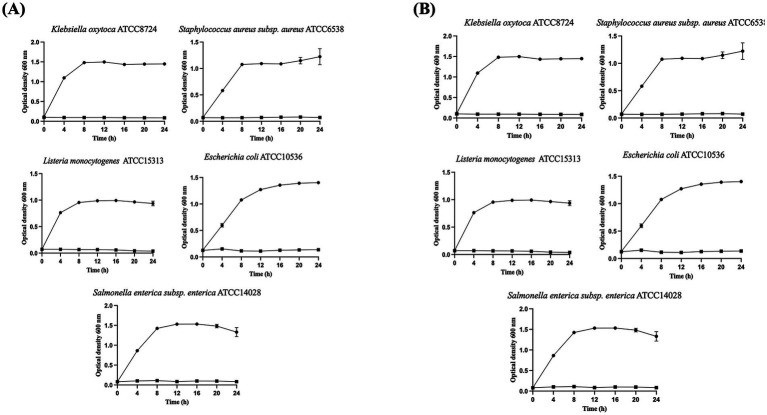
Pathogen growth inhibition by CFS from *W. cibaria* strains. The culture broth of indicator strains was inoculated in a 96-well plate at a final concentration of 1% (v/v) and incubated at 37 °C for 24 h. Black circles, indicator strain growth in LB medium supplemented with 20% (v/v) MRS medium; black squares, indicator strain growth in LB medium supplemented with 20% (v/v) CFS from *W. cibaria* strains. **(A)** Pathogen growth inhibition by CFS from *W. cibaria* LB13201; **(B)** Pathogen growth inhibition by CFS from *W. cibaria* LB13206.

To evaluate antimicrobial potential, an assay using pathogens was conducted. The CFS of *W. cibaria* LB13201 and LB13206 exhibited antimicrobial activity, significantly inhibiting the growth of tested pathogens (Figure 7). The observed suppression of pathogenic bacterial growth appeared to result from organic acids or other non-bacteriocin metabolites produced by the LAB strains. Although typical bacterial growth patterns were followed by the control group, the CFS-treated group showed minimal to no growth, indicating potent antibacterial effects. *W. cibaria* LB13201 exhibited consistent and superior potency, inhibiting all tested pathogens at a volume of 25 μL (Table 4). In contrast, *W. cibaria* LB13206 displayed a differential activity profile; while it was equally effective against *S. aureus* ATCC 6538 (25 μL), it required 50 μL to inhibit *E. coli* ATCC 10536, *K. oxytoca* ATCC 8724, *S. enterica* ATCC 14028, and *L. monocytogenes* ATCC 15313.

**Table 4:** Minimum inhibition concentration of CFS from *W. cibaria* LB13201 and LB13206 against selected pathogenic bacteria.

Bacteria	Cell-free supernatant (μL)	
	LB13201	LB13206
*E. coli* ATCC 10536	25	50
*S. aureus* ATCC 6538	25	25
*K. oxytoca* ATCC 8724	25	50
*S. enterica* ATCC 14028	25	50
*L. monocytogenes* ATCC 15313	25	50

### 3.10 Detection of antibiotic resistance and virulence factor-encoding genes via polymerase chain reaction and minimum inhibitory concentration determination

PCR analysis was conducted to determine whether *W. cibaria* LB13201 and LB13206 harbored any of the 15 antibiotic resistance genes, and none were detected (Supplementary Table S2). Virulence gene analysis confirmed the absence of Aggregation protein (agg), Accessory colonization factor (Ace), Enterococcal surface protein (espA), Endocarditis and Biofilm-associated Pilus (ebp), Cytolysin (cylA), Hyaluronidase (hyI), Gelatinase (gelE), Serine protease (sprE), and Quorum sensing genes (fsrA, fsrB, fsrC) in *W. cibaria* LB13201 and LB13206 genomic sequences (Supplementary Table S2). For *Weissella* spp., EFSA has not yet established cut-off values in antimicrobial susceptibility assessments. Therefore, an antibiotic susceptibility test was conducted following EFSA guidelines for obligate heterofermentative *Lactobacillus* spp. (EFSA et al., 2018). The result confirmed that *W. cibaria* LB13201 and LB13206 were susceptible to all tested antibiotics, including ampicillin, chloramphenicol, gentamycin, clindamycin, streptomycin, erythromycin, kanamycin, and tetracycline (Table 5).

**Table 5:** Minimum inhibitory concentrations and antibiotic susceptibility of *W. cibaria* LB13201 and LB13206.

Antibiotics	MIC (μg/mL)		Cut-off value* (μg/mL)	Susceptibility	Assessment
	LB13201	LB13206			
Ampicillin	0.19	0.19	2	S***	Acceptable
Vancomycin	–	–	n.r.**	S	Acceptable
Gentamycin	1.5	0.19	16	S	Acceptable
Kanamycin	12	8	32	S	Acceptable
Streptomycin	12	8	64	S	Acceptable
Erythromycin	0.064	0.064	1	S	Acceptable
Clindamycin	0.016	0.016	1	S	Acceptable
Tetracycline	1	0.38	8	S	Acceptable
Chloramphenicol	1.5	1	4	S	Acceptable

*Cut-off value is established by the European Food Safety Authority.

**n.r., not required. ***S, Susceptible.

### 3.11 Hemolytic and gelatinase activity assays

When hemolytic activity was assessed on sheep blood agar, *W. cibaria* LB13201 and LB13206 showed no clear zone formation, whereas *S. aureus* subsp. *aureus* ATCC 29213 exhibited a distinct clear zone (Supplementary Figure S2A). Similarly, when cultured on gelatin agar, the medium remained solid and retained the slant shape, indicating the absence of gelatinase activity in *W. cibaria* LB13201 and LB13206. In contrast, *B. subtilis* ATCC 6633 liquefied the gelatin, confirming its proteolytic capability (Supplementary Figure S2B).

### 3.12 Enzymatic activity assessment using the API ZYM kit

The enzymatic profiles of *W. cibaria* strains, assessed using the API ZYM system, were comparable to those reported in previous studies (Muñoz-Atienza et al., 2013). Elevated β-glucuronidase levels in feces are associated with gastric cancer and inflammatory bowel disease (Mroczyńska et al., 2013). Therefore, probiotic strains should not produce carcinogenic enzymes such as β-glucuronidase. *W. cibaria* LB13021 and LB13206 exhibited acid phosphatase and naphthol-AS-BI-phosphohydrolase activity; however, they did not produce β-glucuronidase (Supplementary Table S3).

## 4 Discussion

Comprehensive genomic characterization is essential for validating the safety and efficacy of bacterial strains intended for livestock use. This study analyzed the genomic profiles of *W. cibaria* LB13201 and LB13206, isolated from Hanwoo cattle, to confirm their taxonomic identity and probiotic potential.

ANI analysis robustly supported the classification of both isolates as *W. cibaria*, with all values against publicly available RefSeq genomes exceeding the 95% species delineation threshold (Chambers et al., 2020). LB13201 exhibited its highest ANI (99.63%) with *W. cibaria* AM113-89 (human gut isolate), while LB13206 showed its highest ANI (99.47%) with *W. cibaria* CBA3636 (fermented vegetable isolate). These high ANI values confirm species identity and highlight the genetic conservation within *W. cibaria*, regardless of isolation source.

Despite lacking bacteriocin-encoding genes, both strains exhibited significant antimicrobial activity against *S. aureus* ATCC 6538 and *E. coli* ATCC 10536. This activity correlates with the detection of secondary metabolite biosynthetic gene clusters, specifically T3PKS and terpene-precursor clusters (Figure 2). Unlike proteinaceous bacteriocins, polyketides and terpenes possess broad-spectrum antimicrobial and anti-inflammatory properties (Katsuyama and Ohnishi, 2012), suggesting these metabolites drive the observed antimicrobial effects.

MIC assays quantitatively confirmed this activity. LB13201 demonstrated consistent efficacy, inhibiting all tested pathogens at 25 μL. In contrast, LB13206 displayed a strain-dependent profile, requiring two-fold higher volume to inhibit *L. monocytogenes* ATCC 15313, while maintaining equal effectiveness against *S. aureus* ATCC 6538 (25 μL). When the CFS of LB13201 and LB13206 treated with tested pathogens, typical bacterial growth patterns were followed by the control group, the CFS-treated group showed minimal to no growth, indicating potent antibacterial effects. These antimicrobial properties likely provide a competitive advantage in the gut ecosystem.

The genomic suitability of these strains as feed additives is further supported by their enriched CAZyme profiles, particularly the high abundance of GH13 (starch hydrolysis) and GH43 (hemicellulose degradation) families. Given that Hanwoo cattle feed is rich in plant-based polysaccharides, these enzymes suggest that LB13201 and LB13206 can facilitate nutrient breakdown (Park et al., 2018; Caputo et al., 2021; Liang et al., 2023), potentially enhancing feed efficiency. This enzymatic activity not only improves nutrient accessibility but also generates prebiotic oligosaccharides that selectively promote beneficial bacteria, offering significant health advantages for livestock (Plouhinec et al., 2023). This genomic trait correlates with the strains’ ability to utilize diverse carbon sources, distinguishing them as metabolically versatile probiotics capable of thriving in the complex rumen environment.

Stress response genes identified through genomic analysis provide a molecular basis for the phenotypic robustness observed in acid and bile tolerance assays. Both LB13201 and LB13206 maintained high survival rates (>80%) under bile stress, likely supported by these genetic determinants, ensuring viability during gastrointestinal transit.

A resilient rumen ecosystem requires diverse microbial consortia operating within a favorable pH range of 6.0–7.0 (Mao and Wang, 2025). Lactic acid-producing microbes continuously release lactic acid in the rumen, promoting lactate-metabolizing bacteria activity and contributing to ruminal pH regulation (Dagnaw Fenta et al., 2023). Although LB13201 and LB13206 exhibited limited survival under extremely acidic conditions (pH 2.5–3.0), their strong resistance at pH 4.5 suggests they can survive under physiological rumen conditions and potentially contribute to the rumen ecosystem as probiotics (Fanelli et al., 2022).

The ability to tolerate acid and bile is a critical probiotic prerequisite. Both strains demonstrated excellent resistance to pH 4 and bile salts, with survival rates exceeding 80%, classifying them as having good resistance (Sornplang and Piyadeatsoontorn, 2016). This tolerance is consistent with findings for other *W. cibaria* strains isolated from dairy cows (Patrone et al., 2021) and the general resilience of the *Weissella* genus under acidic conditions (Nami et al., 2024). This may be attributed to stress-response proteins, such as chaperones and proteases, which maintain cellular functions under stress (Patrone et al., 2021; Kandasamy et al., 2022). While survival was limited at pH 2.5–3.0, the strong performance at pH 4.5 is highly relevant for livestock applications, as the typical rumen pH of 6.0–7.0 can create more acidic microenvironments through localized lactic acid production (Dagnaw Fenta et al., 2023; Mao and Wang, 2025; Rodríguez-Sánchez et al., 2021).

Auto-aggregation ability is crucial for gut colonization and creating a protective barrier against pathogens. Both strains demonstrated effective adhesion properties, though with strain-specific differences compared to the reference probiotic *L. rhamnosus* ATCC 53103. Their auto-aggregation rates were comparable to the 18–79% range previously reported for beneficial *Lactobacillus* and *Weissella* species (Rajoka et al., 2017). LB13206 showed co-aggregation with several cattle pathogens at levels similar to or higher than the reference strain, including *K. oxytoca* ATCC 8724, *S. aureus* ATCC 6538, and *L. monocytogenes* ATCC 15313. LB13201 displayed levels mostly similar to or lower than the reference strain, except for its interaction with *S. aureus* ATCC 6538. This process is mediated by cell surface components such as proteins and teichoic acids (Alp and Kuleaşan, 2020), allowing probiotics to directly bind pathogenic bacteria (Lee et al., 2015). Co-aggregation is critical as it enables probiotics to bind and remove pathogens from the gut environment. In cattle health, pathogens like *Klebsiella* spp., *S. aureus*, *L. monocytogenes*, and *E. coli* are major concerns, causing costly diseases such as mastitis and uterine infections (Williams et al., 2008; Kemal, 2014; Massé et al., 2020). Therefore, both strains show potential as probiotic candidates for enhancing gut health and protecting against pathogenic infections in livestock.

The antioxidant properties of LAB are crucial for mitigating oxidative stress caused by free radicals in the host (Ren et al., 2014). Both strains demonstrated notable antioxidant abilities. While antioxidant capacity is a known feature of the *Weissella* genus, the potent activity observed in our strains is comparable to, or exceeds, that reported for other *Weissella* species, suggesting superior potential (Oh et al., 2018). This is significant because oxidative stress is a primary driver of immune dysfunction, particularly during high-demand periods such as the transition phase in dairy cows (Sordillo and Aitken, 2009; Abuelo et al., 2019). Targeted antioxidant supplementation has been shown to enhance mucosal immune responses in calves (Nayak and Abuelo, 2021). Therefore, the antioxidant activity of both strains suggests they could serve as effective feed additives to bolster host defense against oxidative stress and improve health.

An immunomodulatory potential of probiotics is a key functional attribute, but safety must first be established. Both strains exhibited no cytotoxicity in RAW 264.7 macrophage cells, a critical prerequisite for their use. Furthermore, they demonstrated significant anti-inflammatory effects by reducing NO production upon LPS stimulation. This finding is consistent with previous studies where heat-inactivated *W. cibaria* JW15 decreased NO production in the same cell line (Yu et al., 2019b), and *W. cibaria* SDS2.1 down-regulated pro-inflammatory cytokines such as IL-6, IL-1β, and TNF-α in bovine mammary epithelial cells (Ren et al., 2025). Such properties are highly relevant for livestock, as inflammatory conditions like subacute ruminal acidosis are often triggered by LPS from gut bacteria (Uyeno et al., 2015). Given that LPS induces inflammatory mediators like NO via the NF-κB pathway (Choy et al., 2008), the ability of our strains to inhibit NO production suggests potential for modulating this key inflammatory cascade. Therefore, the demonstrated non-cytotoxicity and potent anti-inflammatory activity highlight their promise as feed additives for alleviating inflammation-related disorders in livestock.

Probiotics have been explored as alternatives to conventional antibiotic therapy, which has become less effective due to rising bacterial resistance (Lim et al., 2018). LAB are prominent candidates, known to produce diverse antimicrobial substances, including organic acids, hydrogen peroxide, and bacteriocins (Choi et al., 2018; Šušković et al., 2010). Among LAB, the *Weissella* genus holds significant promise, although research on its bacteriocins remains limited compared to other genera (Han et al., 2024). Early administration of such probiotics to calves can support the establishment of beneficial gut microbiota, thereby reducing the incidence of gastrointestinal infections like diarrhea (Patrone et al., 2021; Uyeno et al., 2015). Based on these antimicrobial properties, both strains are valuable candidates as probiotic feed additives to enhance gastrointestinal health in livestock.

A critical safety assessment for any potential probiotic is its antibiotic resistance profile. The *Weissella* genus generally exhibits susceptible profiles, with only occasional resistance to macrolides or tetracyclines (Fhoula et al., 2022). However, intrinsic resistance to vancomycin is a key characteristic. For instance, *W. koreensis* SK was found to be non-susceptible to vancomycin, reflecting a genus-level phenotype (Mun and Chang, 2020). This trait is recognized as intrinsic in related heterofermentative *Lactobacillus* groups and is not considered a transferable safety risk (EFSA et al., 2018). Consistent with this established pattern, both strains exhibited high MIC values for vancomycin (≥256 μg/mL), aligning with previous reports on this species (Choi et al., 2018; Wang et al., 2018; Kang et al., 2019).

Genomic screening is crucial to confirm the absence of acquired ARG. In a similar study, *W. cibaria* CMU lacked acquired ARG, with most MIC values falling below EFSA-defined cut-offs (Kang et al., 2019). Therefore, the antibiotic resistance profile of LB13201 and LB13206—characterized by intrinsic vancomycin resistance alongside general susceptibility to other antibiotics and no acquired ARG—strongly supports their safety.

A comprehensive safety evaluation requires screening for virulence factors. Genomic analysis confirmed that both strains lack key virulence-associated genes, consistent with the safety profiles of other *W. cibaria* strains. For example, the absence of enterococcal virulence determinants (*e*sp.*, gelE*, *sprE*, *cylA*, *hyl*, *ace*, *efaA*) was reported in *W. cibaria* W21, W25, and W42 (Teixeira et al., 2024), and no acquired virulence genes were found in the kimchi-derived strain *W. cibaria* JW15 (Jang et al., 2021).

Specifically, the absence of genes implicated in biofilm formation and adhesion, such as *ebp* and *esp*., suggests diminished capacity for establishing persistent infections (Nallapareddy et al., 2006). The lack of *cylA* and *hyl* indicates reduced potential for hemolysis and tissue invasion, corroborating the non-hemolytic phenotypes repeatedly observed in *W. cibaria* (Kang et al., 2019; Jang et al., 2021). Particularly important is the absence of the fsr quorum-sensing system (*fsrA*, *fsrB*, *fsrC*), a key regulator of virulence factor expression in pathogens like *Enterococcus faecalis* (Qin et al., 2000). The absence of recognized virulence factors in both strains provides strong genomic evidence supporting their classification as safe microorganisms. A comprehensive safety assessment necessitates the evaluation of key virulence-associated activities. Both strains have been confirmed as non-hemolytic, indicating that they do not produce toxins capable of lysing red blood cells. This is a fundamental safety requirement outlined by EFSA guidelines and is consistent with previous reports for other *W. cibaria* strains, reinforcing the generally safe profile of this species (Kang et al., 2019; Yasmin et al., 2020).

Furthermore, both strains tested negative for gelatinase activity. Gelatinase is a proteolytic enzyme considered a significant virulence factor due to its ability to degrade connective tissues, facilitating tissue invasion and pathogenesis (Mortezaei et al., 2021). The absence of this enzyme indicates reduced potential for harmful proteolytic activity within a host.

Enzymatic profile is crucial for determining metabolic capabilities and safety. Using the API ZYM system, both strains exhibited beneficial enzyme activities, including acid phosphatase and naphthol-AS-BI-phosphohydrolase. The presence of phosphatases is desirable for probiotics, as these enzymes can hydrolyze anti-nutritional compounds like phytic acid in feed, thereby increasing the bioavailability of essential minerals (Joudaki et al., 2023).

Both strains were negative for β-glucuronidase activity. Previous reports show that strains negative for β-glucuronidase are suitable for safe probiotic use, as this enzymatic activity is associated with harmful intestinal effects (Bujnakova et al., 2014). This enzymatic profile, characterized by beneficial enzymes and the absence of harmful ones, is consistent with previous reports for *W. cibaria* (Muñoz-Atienza et al., 2013) and further supports the classification of LB13201 and LB13206 as both safe and functionally promising for probiotic applications. *W. cibaria* LB13201 and LB13206 isolated from Hanwoo cattle exhibited several functional attributes associated with probiotic potential, including acid and bile salt tolerance, antioxidative and anti-inflammatory activities, and antimicrobial effects. Whole-genome sequencing has supported the taxonomic classification of the strains, indicating an absence of virulence or antibiotic resistance genes. Additionally, susceptibility to all tested antibiotics was demonstrated, suggesting their safety as feed additives. These results indicate that both strains are promising candidates for enhancing gastrointestinal health and suppressing pathogens in animal hosts. Further investigations, including *in vivo* studies in relevant animal models, will be necessary to evaluate their functional efficacy and safety under practical feeding conditions.

## Data Availability

The whole-genome sequence data for the strains used in this study have been deposited in the NCBI GenBank database. The accession numbers are JBSUSF000000000.1 for Weissella cibaria LB13201 and JBSUSG000000000.1 for Weissella cibaria LB13206.

## Footnotes

1
https://cge.food.dtu.dk/services/ResFinder

2
https://card.mcmaster.ca/

3
https://cge.food.dtu.dk/services/VirulenceFinder

4
http://www.graphpad.com
