# Supplementary material for: Genomic and phenotypic analysis of Weissella cibaria LB13201 and LB13206 isolated from Hanwoo (native Korean cattle) with antimicrobial and anti-inflammatory capability
Source: Front Microbiol. 2026 Feb 19;17:1674601. doi: 10.3389/fmicb.2026.1674601 (PMC12960619; doi:10.3389/fmicb.2026.1674601)
Supplement: Supplementary file 1 [file Supplementary_file_1.docx]

Supplementary Material

# Supplementary Figures and Tables

## Supplementary Figures


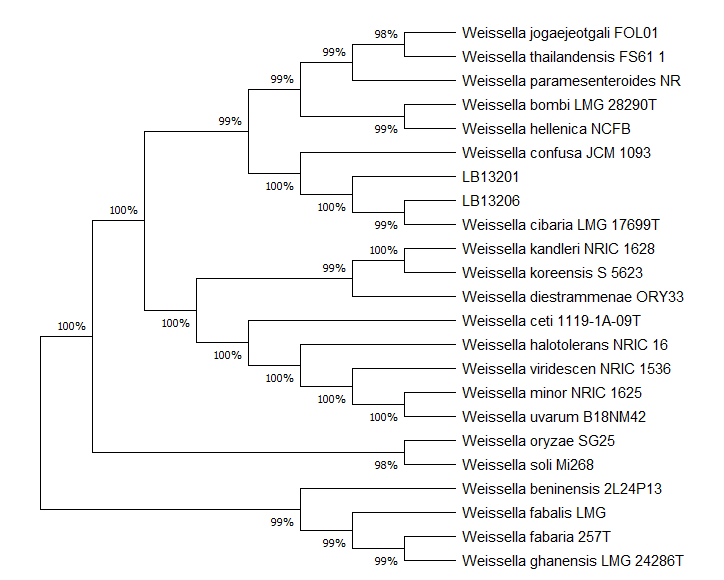


**Supplementary Figure 1.** Phylogenetic tree of *W. cibaria* LB13201 and LB13206.


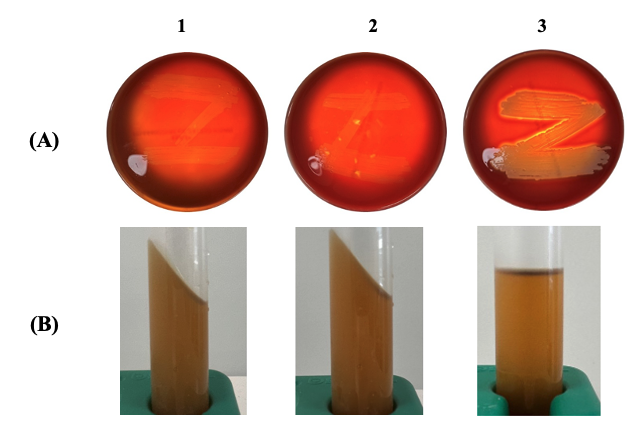


**Supplementary Figure 2.** The Hemolysis test using blood agar plate and gelatinase test using gelatin agar. (A) Hemolysis test; (B) gelatin liquefaction test using MRS medium containing 12% gelatin by 1. *W. cibaria* LB13201, 2. *W. cibaria* LB13206, and 3. positive control (A, *S. aureus* subsp. *aureus* ATCC 29213 B, *B. subtilis* ATCC 6633).


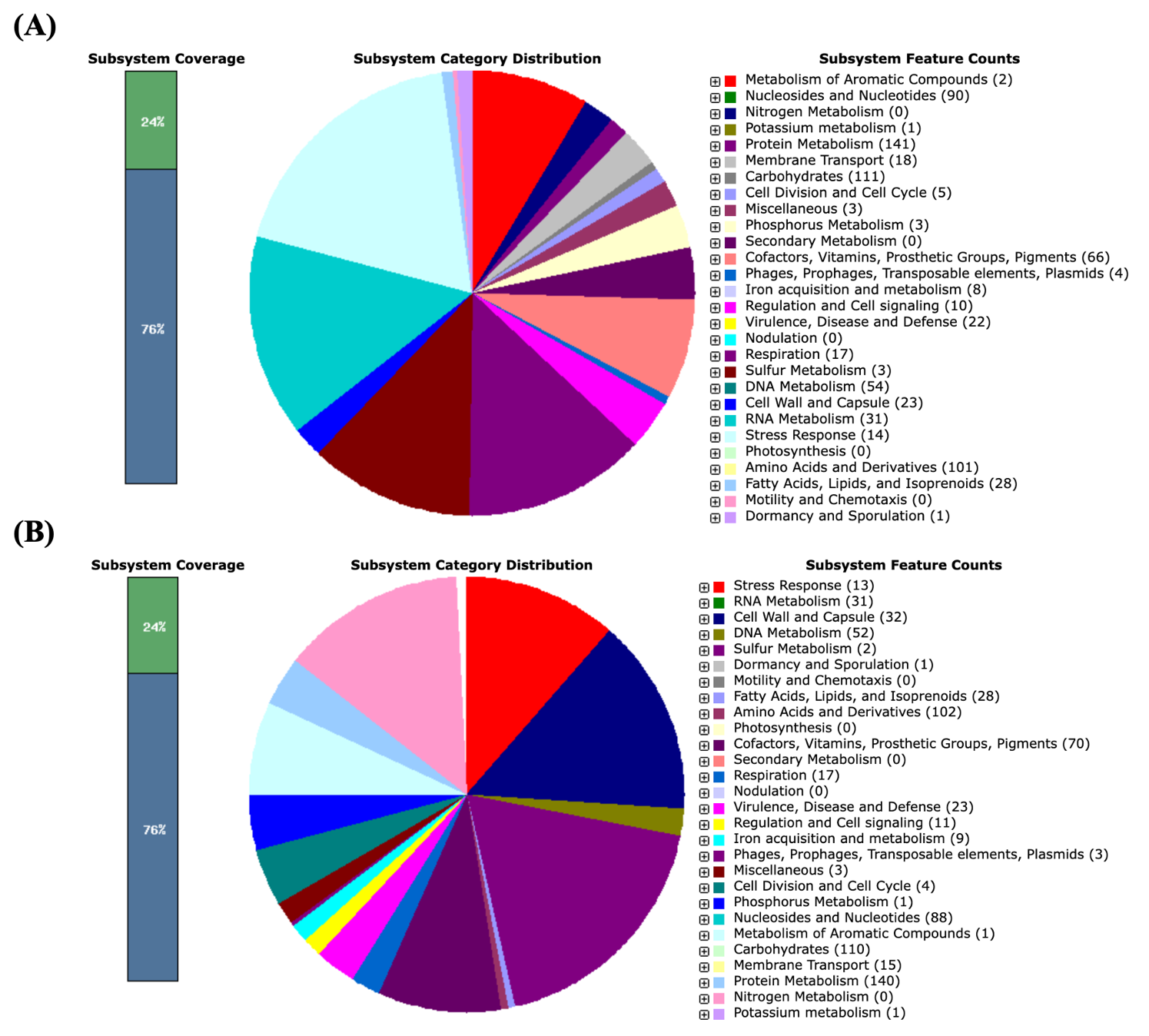


**Supplementary Figure 3.** Subsystem-based functional classification of annotated genes and subsystem feature counts generated using the RAST (Rapid Annotations using Subsystems Technology) server. (A) *W. cibaria* LB13201; (B) *W. cibaria* LB13206.

## Supplementary Tables

Supplementary Table 1 Gene-specific primers and conditions for PCR detection.

| **Antibiotics & Virulence factor** | **Target gene** | **Primer sequence (5′ → 3′)** | **Annealing temperature (°C)** | **Target size (bp)** | **References** |
| --- | --- | --- | --- | --- | --- |
| Ampicillin | *bla* | CATATRTTCGCATAATASMGCC | 48 | 297 | (Kang et al., 2019) |
|  |  | CGTSTTAACTAAGTATSGY |  |  |  |
| Chloramphenicol | *cat* | TTAAGGTTATTGGCAGTAGTTA | 50 | 300 | (Kang et al., 2019) |
|  |  | GCATGRATACCATAACAWAC |  |  |  |
| Erythromycin | *erm*(B) | CATTTAGACCAGGAACATCGGC | 60 | 640 | (Kang et al., 2019) |
|  |  | GGAACATCTGGTGTAATGACG |  |  |  |
|  | *erm*(c) | CAAACCGCATGGTATGGCAATT | 60 | 295 | (Kang et al., 2019) |
|  |  | ATCTTTAAGCCATATCATGTTTG |  |  |  |
| Gentamicin | *aac*(6'*)*-*aph*(2'*)* | CAGAAGACATGACGGATTGG | 52 | 220 | (Kang et al., 2019) |
|  |  | CACTATCATAACCACTACCAG |  |  |  |
| Kanamycin | *aph*(3')-III | GCCGATTGCGATATGGGAAAG | 60 | 292 | (Kang et al., 2019) |
|  |  | GCTTGATCCCGAATGAGTCA |  |  |  |
| Streptomycin | *str*A | CTGGTGTAAGGTCACGAGTTC | 60 | 548 | (Aristimuño Ficoseco et al., 2018) |
|  |  | CCAAGCTCTGCCAGGCAAGTC |  |  |  |
|  | *str*B | ATCTGCAAGGAGATTGCGG | 57 | 509 | (Aristimuño Ficoseco et al., 2018) |
|  |  | GGATCGTCAAGGTTCTCCAG |  |  |  |
|  | *aad*A | ATCTCTTGCGATTTCGCCATCGTTG | 65 | 282 | (Kang et al., 2019) |
|  |  | GCAATCCTTCACCTTCGG |  |  |  |
|  | *aad*E | ATGGGATTATTTCCCCGAT | 57 | 565 | (Kang et al., 2019) |
|  |  | TGCAAACCCAAACCAATCCG |  |  |  |
|  | *ant*(6) | ACTGGCTACATCGATAGG | 60 | 597 | (Kang et al., 2019) |
|  |  | CGGTTTCCGCCAGTTCACGC |  |  |  |
| Tetracycline | *tet*(M) | GTGGTAAAGGCAATGCACGAG | 57 | 406 | (Aristimuño Ficoseco et al., 2018) |
|  |  | CGCATACCTGATTTGCGG |  |  |  |
|  | *tet*(K) | GTGCGATTGAGCCTTCTG | 57 | 697 |  |
|  |  | CGAATCATGGCTCAGGCG |  |  |  |
|  | *tet*(L) | CATTTCAGGTTTTGTTGATG | 57 | 456 |  |
|  |  | ATTACACATCCAGTCTTACT |  |  |  |
|  | *tet*(S) | TGAACACGCCAGGACAAATT | 57 | 660 |  |
|  |  | ACATAGACAGGCGTTGACC |  |  |  |
| Aggregation protein | *Agg* | AAAGAAAGAAGTAGCACCAAC | 53 | 1553 | (Aristimuño Ficoseco et al., 2018) |
|  |  | AAACCGCAAGTGCAATAATA |  |  |  |
| Accessory colonization factor | *Ace* | CAGAGCAAACGAGTGCTAAA | 65 | 1553 | (Aristimuño Ficoseco et al., 2018) |
|  |  | AACCTAGTTTGGTTCAAGTT |  |  |  |
| Enterococcal surface protein | *Esp*A | TTTGCGGCAAGTGCGAATAGT | 60 | 125 | (Aristimuño Ficoseco et al., 2018) |
|  |  | CCCAGCAAGTCGATCACTAT |  |  |  |
| Endocarditis and Biofilm-associated Pilus | *ebp* | AATGTGTTTAGCTTGAACACTG | 62 | 407 | (Aristimuño Ficoseco et al., 2018) |
|  |  | ACTCCTTTGGAAGTGACACAT |  |  |  |
| Cytolysin | *cyl*A | ACTCGGCGATTAGTGAAGGC | 60 | 372 | (Aristimuño Ficoseco et al., 2018) |
|  |  | GCTCGTAAGTAGTTCATCATTC |  |  |  |
| Hyaluronidase | *Hyl* | ACAGAGAGCAGCTCGAAGAATG | 62 | 688 | (Aristimuño Ficoseco et al., 2018) |
|  |  | GACTCAGAGATCGTGCTCCAA |  |  |  |
| Gelatinase | *gel*E | CGAAGTTGGTGAAGACAGAGGC | 50 | 276 | (Aristimuño Ficoseco et al., 2018) |
|  |  | GGTGAACAGTTCGTACTCGA |  |  |  |
| Serineprotease | *spr*E | GTGTAACCGCAGTGAGTGC | 57 | 372 | (Aristimuño Ficoseco et al., 2018) |
|  |  | TTCTCCTACTTCTGGCTGGC |  |  |  |
| Quorum sensing genes | *fsr*A | TGATGAAGTGTGATTAGGAC | 60 | 300 | (Aristimuño Ficoseco et al., 2018) |
|  |  | ATTACAGGTTGAGTTGGCAC |  |  |  |
|  | *fsr*B | TGGACAAACTTGACCTATGGC | 57 | 744 |  |
|  |  | CACACCATCACTAGTTTTGC |  |  |  |
|  | *fsr*C | ATCAGACTCAGATCCCAATAAGC | 52 | 729 |  |
|  |  | ACGACTATTCAGTGTTAAAGC |  |  |  |

Supplementary Table 2 PCR detection of antibiotic resistance and virulence factor genes in W. cibaria strains.

| **Antibiotics & Virulence factor** | **Target gene** | ***W. cibaria* LB13201** | ***W. cibaria* LB13206** |  |
| --- | --- | --- | --- | --- |
| Ampicillin | *bla* | - | - |  |
| Chloramphenicol | *cat* | - | - |  |
| Erythromycin | *erm*(B) | - | - |  |
|  | *erm*(C) | - | - |  |
| Gentamicin | *aac*(6')-*aph*(2') | - | - |  |
| Kanamycin | *aph*(3')-III | - | - |  |
| Streptomycin | *str*A | - | - |  |
|  | *str*B | - | - |  |
|  | *aad*A | - | - |  |
|  | *aad*E | - | - |  |
|  | *ant*(6) | - | - |  |
| Tetracycline | *tet*(M) | - | - |  |
|  | *tet*(K) | - | - |  |
|  | *tet*(L) | - | - |  |
|  | *tet*(S) | - | - |  |
| Aggregation protein | *Agg* | - | - |  |
| Accessory colonization factor | *Ace* | - | - |  |
| Enterococcal surface protein | *esp*A | - | - |  |
| Endocarditis and Biofilm-associated Pilus | *ebp* | - | - |  |
| Cytolysin | *cyl*A | - | - |  |
| Hyaluronidase | *Hyl* | - | - |  |
| Gelatinase | *gel*E | - | - |  |
| Serineprotease | *spr*E | - | - |  |
| Quorum sensing genes | *fsr*A | - | - |  |
|  | *fsr*B | - | - |  |
|  | *fsr*C | - | - |  |

*–, not detected

Supplementary Table 3 API zym test result of W. cibaria strains.

| **Enzyme** | ***W. cibaria* LB13201** | ***W. cibaria* LB13206** |
| --- | --- | --- |
| Alkaline phosphatase | - | - |
| Esterase (C4) | - | - |
| Esterase Lipase(C8) | - | - |
| Lipase(C14) | - | - |
| Leucine arylamdiase | - | - |
| Valine arylamdiase | - | - |
| Crystine arylamdiase | - | - |
| Trypsin | - | - |
| α-chymotrypsin | - | - |
| Acid phospatase | + | + |
| Naphtol-AS-Bl-phosphohydrolase | + | + |
| α-galactosidase | - | - |
| β-glucuronidase | - | - |
| β-glucosidase | - | - |
| α-glucosidase | - | - |
| β-glucosidase | - | - |
| N-acetyl-β-glucosaminidase | - | - |
| α-mannosidase | - | - |
| α-fucosidase | - | - |

*+, detected; –, not detected
